# Salvage endoscopic submucosal resection for residual esophageal superficial cancer involving a stenotic anastomosis: a challenging but desirable indication

**DOI:** 10.1055/a-2106-1096

**Published:** 2023-07-13

**Authors:** Jiyu Zhang, Huige Wang, Miao Shi, Dan Liu, Bing-Rong Liu

**Affiliations:** Department of Gastroenterology and Hepatology, The First Affiliated Hospital of Zhengzhou University, Zhengzhou, China

A 48-year-old man with a history of neoadjuvant chemoradiotherapy followed by subtotal esophagectomy presented with symptoms of dysphagia over 2 months. Endoscopic examination showed an anastomotic stenosis at 20 cm from the incisors and the endoscope was unable to be passed through this ( Fig. 1 a ). In addition, white-light endoscopy and narrow-band imaging revealed a patch of residual early esophageal cancer, with meandering vessels, in a pseudodiverticulum near the anastomosis ( Fig. 1 b ). A salvage endoscopic submucosal dissection (ESD) was scheduled to remove this lesion, with the aim of avoiding its further malignant progression and aggressive additional treatment ( Video 1 ). Nonstaining of the lesion with Lugol's iodine chromoendoscopy helped to delineate the margin ( Fig. 1 c ). Intraoperatively, although submucosal lifting was not satisfactory because of the marked fibrosis, it was still possible to complete en bloc dissection ( Fig. 1 d ).

**Fig. 1:**
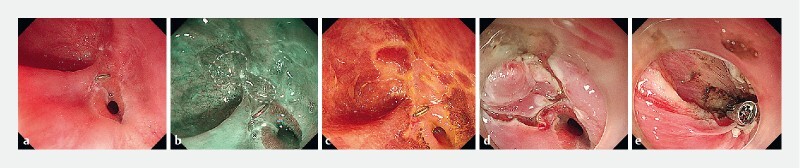
Endoscopic images showing: **a** the stenotic anastomosis; **b** on narrow-band imaging, a patch of abnormal mucosa with a clearly delineated margin in a pseudodiverticulum near the anastomosis; **c** the boundary clearly delineated on Lugol’s iodine chromoendoscopy; **d** the appearance after endoscopic submucosal dissection had been performed; **e** the post-endoscopic submucosal dissection defect and a clip in place after a longitudinal incision of the anastomosis had been made.

**Video 1** Salvage endoscopic submucosal resection is performed for residual esophageal superficial cancer involving a stenotic anastomosis after chemoradiotherapy and subtotal esophagectomy.


Furthermore, an endoscopic longitudinal incision was performed to relieve the anastomotic stenosis. After incision of the anastomosis, an endoscopic clip was used to bridge the opposing mucosa of both anastomotic edges ( Fig. 1 e ), and the endoscope was able to pass through the narrowed segment smoothly after the procedure. The final pathology result revealed a high grade glandular intraepithelial neoplasm, with R0 resection ( Fig. 2 ). The patient was discharged on postoperative day 4, with no adverse events having occurred.

**Fig. 2:**
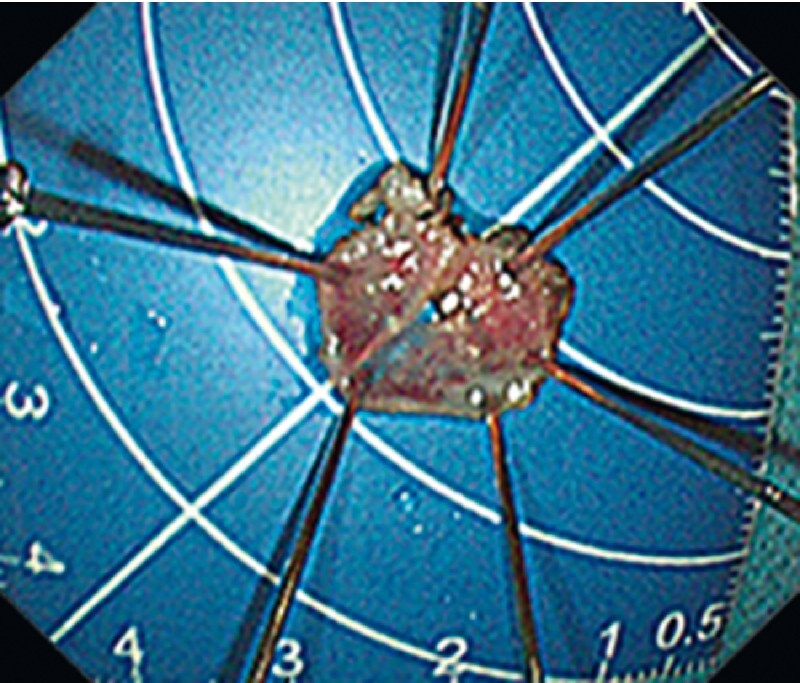
Macroscopic appearance of the resected lesion.

Locoregional recurrence or residue remains the major cause of failure, occurring in 50 %–75 % of patients treated with surgery and/or chemoradiotherapy for esophageal cancer 1 2 . The scar tissue at the anastomotic site becomes rigid, resulting in poor lifting, so surgical reoperation of such lesions is technically more challenging and can cause complications 3 . ESD has been widely used for superficial esophageal cancer 4 5 . Although ESD of a lesion involving a surgical anastomosis and pseudodiverticulum is challenging, this salvage treatment still offers significant clinical advantages in experienced hands.

Endoscopy_UCTN_Code_CCL_1AB_2AC_3AB

## Notes

Endoscopy E-Videos

https://eref.thieme.de/e-videos
*E-Videos* is an open access online section of the journal *Endoscopy* , reporting on interesting cases and new techniques in gastroenterological endoscopy. All papers include a high-quality video and are published with a Creative Commons CC-BY license. Endoscopy E-Videos qualify for HINARI discounts and waivers and eligibility is automatically checked during the submission process. We grant 100% waivers to articles whose corresponding authors are based in Group A countries and 50% waivers to those who are based in Group B countries as classified by Research4Life (see: https://www.research4life.org/access/eligibility/ ). This section has its own submission website at https://mc.manuscriptcentral.com/e-videos
